# Optimal vocal therapy for respiratory muscle activation in patients with COPD: effects of loudness, pitch, and vowels

**DOI:** 10.3389/fphys.2024.1496243

**Published:** 2025-01-13

**Authors:** Zhengtong Qiao, Ziwei Kou, Jiazhen Zhang, Daozheng Lv, Dongpan Li, Xuefen Cui, Kai Liu

**Affiliations:** ^1^ School of Special Education and Rehabilitation, Binzhou Medical University, Yantai, China; ^2^ School of Clinical Medicine, Qingdao University, Qingdao, China; ^3^ School of Sports and Health, Shandong Sport University, Jinan, China; ^4^ Department of Respiratory and Critical Medicine, Qingdao Municipal Hospital, Qingdao, China; ^5^ Department of Rehabilitation Medicine, Qingdao Municipal Hospital, University of Health and Rehabilitation Sciences, Qingdao, China

**Keywords:** chronic obstructive pulmonary disease, vocalization training, neural respiratory drive, respiratory muscle, surface electromyographic

## Abstract

**Background:**

Vocal therapy, such as singing training, is an increasingly popular pulmonary rehabilitation program that has improved respiratory muscle status in patients with chronic obstructive pulmonary disease (COPD). However, variations in singing treatment protocols have led to inconsistent clinical outcomes.

**Objective:**

This study aims to explore the content of vocalization training for patients with COPD by observing differences in respiratory muscle activation across different vocalization tasks.

**Methods:**

All participants underwent measurement of surface electromyography (sEMG) activity from the sternocleidomastoid (SCM), parasternal intercostal muscle (PARA), seventh intercostal muscle (7thIC), and rectus abdominis (RA) during the production of the vowels/a/,/i/, and/u/at varying pitches (comfortable, +6 semitones) and loudness (−10 dB, +10 dB) levels. The Visual Analog Scale (VAS) was used to evaluate the condition of patients concerning vocalization, while the Borg-CR10 breathlessness scale was utilized to gauge the level of dyspnea following the task. Repeated-measure (RM) ANOVA was utilized to analyze the EMG data of respiratory muscles and the Borg scale across different tasks.

**Results:**

Forty-one patients completed the experiment. Neural respiratory drive (NRD) in the SCM muscle did not significantly increase at high loudness levels (VAS 7-8) compared with that at low loudness levels (*F* (2, 120) = 1.548, *P* = 0.276). However, NRD in the PARA muscle (*F* (2, 120) = 55.27, *P*< 0.001), the 7thIC muscle (*F* (2, 120) = 59.08, *P* < 0.001), and the RA muscle (*F* (2, 120) = 39.56, *P* < 0.001) were significantly higher at high loudness compared with that at low loudness (VAS 2-3). Intercostal and abdominal muscle activation states were negatively correlated with maximal expiratory pressure (r = −0.671, *P* < 0.001) and inspiratory pressure (r = −0.571, *P* < 0.001) in the same loudness.

**Conclusion:**

In contrast to pitch or vowel, vocal loudness emerges as a critical factor for vocalization training in patients with COPD. Higher pitch and loudness produced more dyspnea than lower pitch and loudness. In addition, maximal expiratory/inspiratory pressure was negatively correlated with respiratory muscle NRD in the same loudness vocalization task.

## 1 Introduction

Chronic obstructive pulmonary disease (COPD) is a heterogeneous lung condition characterized by chronic respiratory symptoms (dyspnea, cough, sputum production, and/or exacerbations) due to abnormalities of the airways and/or alveoli that cause persistent airflow obstruction (Celli et al., 2022). According to the estimation of large-scale epidemiology research, the global epidemic rate of COPD is 10.3% [95% confidence interval (CI) = 8.2%–12.8%], with the progress of the global population aging, the prevalence of chronic obstructive pulmonary disease will continue to increase (Adeloye et al., 2022; Adeloye et al., 2015). Although pulmonary damage in COPD is permanent, symptoms, such as respiratory muscle weakness and dyspnea, can be improved through pulmonary rehabilitation (Nolan et al., 2022).

Vocalization, such as singing, as a pulmonary rehabilitation program can combine specific abdominal respiratory patterns with respiratory muscle training to provide positive expiratory pressure and improve lung dynamic suction (O'Donnell and Webb, 2008; Kaasgaard et al., 2022; McNamara et al., 2017). Vocalization training can enhance exhalation muscle strength and FEV_1_ in patients with COPD(McNamara et al., 2017; Bonilha et al., 2009). However, variations in vocalization treatment protocols have led to inconsistent clinical outcomes (Lord et al., 2012). Developing and applying effective vocalization mechanisms face challenges, including lack of consistent research content and a standardized vocalization protocol (Fang et al., 2022).

In addition, studies have shown significant differences in subglottal pressure during the vocalization of different vowels, suggesting that vowels may produce different afterloads in respiratory muscles (Pettersen, 2005). On the other hand, the activation of respiratory muscles varies with different loudness and pitch vocalization levels (Wang and Yiu, 2023). Hence, pitch, loudness, and vowels may be the main factors that contribute to differences in respiratory muscle activation during vocal content (Higgins, Netsell, and Schulte, 1998). In addition, unlike relaxed, natural expiration, vocalization requires the coordination of expiratory muscles and tends to cause dyspnea (Pettersen and Westgaard, 2004). Respiratory muscles, such as abdominal muscles, are the driving force for the power of vocalization. Although it is usually considered an inspiratory muscle, SCM is activated during speech (Wang and Yiu, 2023). Neural respiratory drive (NRD) measured by surface electromyography (sEMG) is a noninvasive measure of respiratory muscle activation that can be used in studies of physiologic mechanisms of respiratory muscles (Lin et al., 2019; Suh et al., 2020; AbuNurah, Russell, and Lowman, 2020).

Understanding the NRD of respiratory muscles in different vocalization tasks helps develop vocalization training programs for patients with COPD (Jolley et al., 2015; Pozzi et al., 2022). We aimed to 1) monitor the NRD of the SCM, PARA, 7thIC, and RA during various vocalization tasks by using sEMG; 2) evaluate the dyspnea index across different vocalization tasks; and 3) identify factors associated with respiratory muscle activation. We hypothesized that target muscles are more activated at high pitch and loudness and show different activity levels in vowel control tasks.

## 2 Materials and methods

From April 2023 to June 2023, we conducted a non-blind observational study in Qingdao, China. This study was registered at ChinaTrials.gov under the identifier ChiCTR2100052874 and was approved by the Ethics Committee of Qingdao Municipal Hospital. We posted the COPD pulmonary rehabilitation poster in several medical facilities to recruit participants. We screened other patients referred to outpatient clinics for pulmonary rehabilitation to determine their eligibility. The observation experiment occurred after formal recruitment in the intervention study, during their pulmonary rehabilitation sessions. Before participation, all patients were provided informed consent by signing a written document confirming their complete understanding of the study’s purpose, procedures, and potential risks or benefits. The inclusion criteria for study patients encompassed a diagnosis of COPD based on the Global Initiative for Chronic Obstructive Lung Disease (GOLD), willingness to participate in the group, and normal vocal function. Patients with unstable heart diseases or severe cognitive impairment were excluded from the study.

### 2.1 Experimental protocol

After instructing patients in vocal techniques (pitch and loudness), the vowels of /a/,/i/, and/u/were assessed at varying pitch and loudness levels through a visual analog scale (VAS). Task1: To observe different vocal pitches, patients were instructed to relax their whole body and pronounce /a/,/i/, and/u/at low (VAS 2-3) and high pitches (VAS 7-8) with the same loudness (Bane et al., 2023; Awan et al., 2013; Awan et al., 2012). Task 2: To observe different vocal loudness, patients were instructed to pronounce /a/,/i/, and/u/at low (VAS 2-3) and high loudness (VAS 7-8) with the same pitches. Task 3: The same vocalization loudness of 60 dB was selected, and the patient was instructed to continue for more than 5 s to reach the tension-time threshold. The visual feedback interface displays real-time loudness. The patients had rest time between tasks and communicate fully to one another to ensure that patients are relaxed before pronouncing. The Borg-CR10 breathlessness scale was used to assess the degree of dyspnea of the patients after each cycle of /a/,/i/,/u/. Two tasks were carried out at different periods, and the patients were assured of adequate rest before each task. A decibel meter measured at least 15 dB between low and high levels for accurate loudness difference. To assess different pitches, the patients initially produced a comfortable pitch while maintaining a comfortable loudness and then shifted to a higher base pitch (at least +5 semitones) while monitoring the pitch by using online tuning software (Wang and Yiu, 2023). Throughout the process, the voice was required to maintain a stable loudness and pitches and measured approximately 20 cm away from the participant. Data acquisition concluded when the voice reached a weak level (decrease of more than 5 dB or 2 semitones).

### 2.2 Surface EMG protocol

**FIGURE 1:**
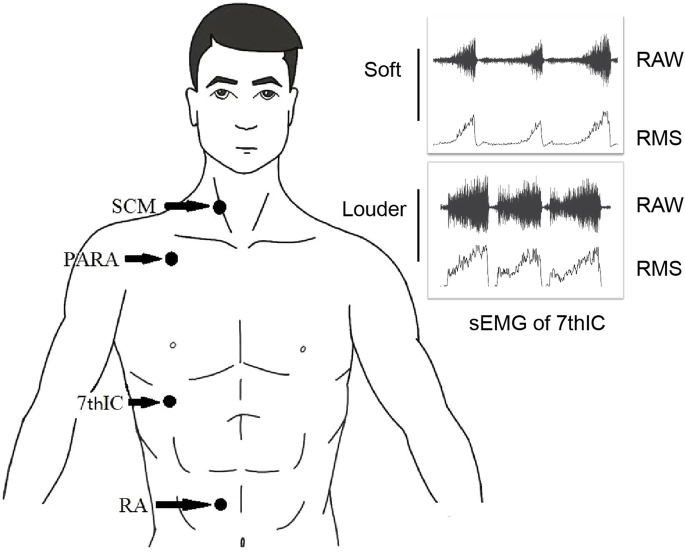
Electrode position and sEMG signals processing. Abbreviations: SCM, sternocleidomastoid; PARA, parasternal intercostal muscle; 7thIC, seventh intercostal muscle; RA, rectus abdominis; RAW, raw surface electromyographic signal; RMS, root-mean-square of surface electromyographic signal.

The Delsys Trigno™ wireless system (Delsys, Natick, MA) and four attached electrodes captured EMG signals at a sampling rate of 2,000 Hz. Wireless electrodes were placed at specific anatomical locations to ensure accurate measurements: the middle and lower 1/3 of the SCM, the junction of the PARA, the 7thIC, and 3 cm above the umbilicus in the position of the RA (Figure 1). Before electrode placement, the skin was meticulously cleaned with a medical alcohol pad to ensure optimal signal acquisition (Liu et al., 2019; da Fonsêca et al., 2019; Cabral et al., 2018). All surface EMG electrodes were positioned on the right side of the body for consistency (Ramsook et al., 2016). After data acquisition, all sEMG signals were analyzed using EMG works analysis software (Delsys, Natick). The sEMG signals recorded were filtered by a 20–450 Hz band-pass Butterworth filter. The signals were then segmented using a root-mean-square (RMS) value calculated based on a 100 ms moving window (Stepp, 2012) (Figure 1). The sEMG signal was calibrated as a percentage of the sEMG signal at maximum voluntary contraction (MVC) (Nguyen et al., 2020). NRD was used to represent RMS%MVC. During the performance of the MVC task, the subjects were instructed to exert their best effort by conducting three maximum breath tests (Cabral et al., 2018).

### 2.3 Statistical analysis

Statistical analysis used SPSS software v26.0 and GraphPad Prism v9.5.1. Data with normal distribution were presented as mean ± standard deviation (SD), while data with a skewed distribution were expressed as median ± interquartile range (IQR). Repeated-measures analysis of variance (RM-ANOVA) was utilized to analyze the EMG data of respiratory muscles and Borg scale across different tasks. Correlation analysis was conducted using Pearson correlation. A 95% confidence interval was established, and the significance level was set at 0.05.

## 3 Results

Forty-eight patients who met the criteria were recruited and trained; five were excluded because they could not maintain pitch or loudness, and two were excluded because they could not support sound. Finally, forty-one patients successfully concluded the experiment. No adverse events occurred during the experiment. Demographic and anthropometric data of patents with COPD patients who underwent voice tasks are shown in Table 1.

**TABLE 1:** Basic information of subjects.

Subjects	41
Age (year)	66.3 (6.0)
Sex	
Male (%)	32 (78.0%)
Female (%)	9 (22.0%)
Height (m)	1.7 (0.1)
Weight (kg)	75.2 (12.5)
BMI (kg·m^−2^)	26.0 (3.7)
Pack years	40 (10)
FEV_1_%pred	50.3 (15.0)
GOLD classification	
Class 1 (%)	8 (19.5%)
Class 2 (%)	22 (53.6%)
Class 3 (%)	11 (26.8%)
Class 4 (%)	0
MEP (mmHg)	68.7 (17.7)
MIP (mmHg)	81.0 (21.6)

Note: Data are presented as median (IQR) or n (%) unless otherwise stated; BMI, body mass index; FEV_1_% pred, forced expiratory volume in one second % predicted; GOLD, global initiative for chronic obstructive lung disease criteria; MEP, maximal expiratory pressure; MIP, maximal inspiratory pressure.

### 3.1 NRD of muscles in pitch control

We found no difference at the NRD of SCM (*F* (2, 120) = 0.116, *P* = 0.890), PARA (*F* (2, 120) = 0.034, *P* = 0.967), 7thIC (*F* (2, 120) = 0.755, *P* = 0.473), and RA (*F* (2, 120) = 0.019, *P* = 0.982) during the high pitches task compared with the low pitch task (Table 2).

**TABLE 2:** Comparison of muscle activation in different task states.

Muscle/Task	Detailed characterisation of Tasks			*F*	*P*
Pitch control	Low	Medium	High		
SCM	0.46 (0.14)	0.47 (0.10)	0.47 (0.14)	0.116	0.890
PARA	0.48 (0.11)	0.47 (0.09)	0.47 (0.14)	0.034	0.967
7thIC	0.54 (0.10)	0.53 (0.07)	0.51 (0.09)	0.755	0.473
RA	0.51 (0.08)	0.51 (0.07)	0.51 (0.10)	0.019	0.982

Loud control	Soft	Medium	Louder		
SCM	0.43 (0.13)	0.47 (0.10)	0.46 (0.14)	1.300	0.276
PARA	0.37 (0.10)	0.59 (0.11)	0.47 (0.09)	55.27	**<0.001** ^a^
7thIC	0.41 (0.10)	0.53 (0.07)	0.63 (0.10)	59.08	**<0.001** ^a^
RA	0.41 (0.09)	0.50 (0.08)	0.58 (0.10)	39.56	**<0.001** ^a^

Vowel control	/a/	/i/	/u/		
SCM	0.46 (0.09)	0.46 (0.09)	0.47 (0.10)	0.309	0.735
PARA	0.48 (0.08)	0.47 (0.08)	0.47 (0.07)	0.058	0.944
7thIC	0.52 (0.08)	0.52 (0.07)	0.52 (0.07)	0.051	0.950
RA	0.52 (0.08)	0.50 (0.07)	0.49 (0.07)	2.128	0.124

Note: SCM, sternocleidomastoid; PARA, parasternal intercostal muscle; 7thIC, seventh intercostal muscle; RA, rectus abdominis.

^a^ *P*< 0.05 indicates a statistical difference.

### 3.2 NRD of muscles in loudness control

**FIGURE 2:**
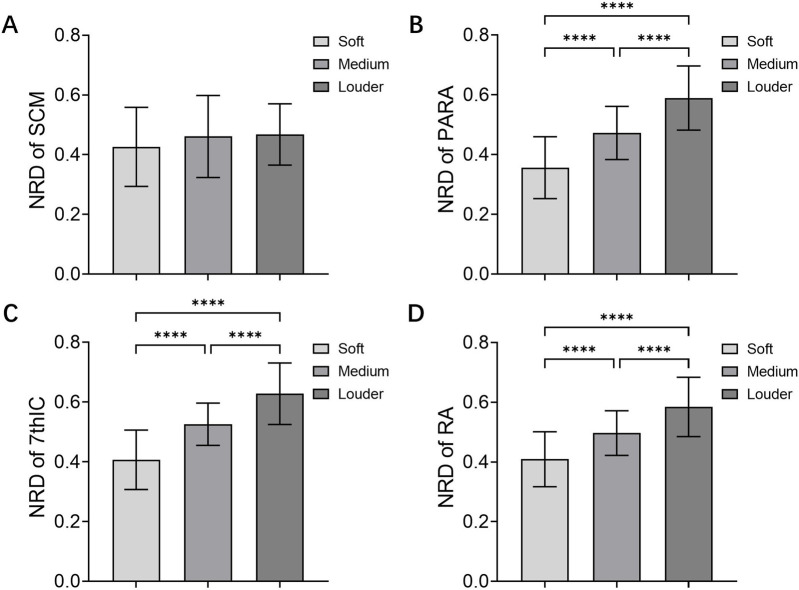
NRD of Muscles in loudness control. NRD of **(A)** SCM, **(B)** PARA, **(C)** 7thIC, and **(D)** RA during soft and louder loudness. Abbreviations: NRD, neural respiratory drive; SCM, sternocleidomastoid; PARA, parasternal intercostal muscle; 7thIC, seventh intercostal muscle; RA, rectus abdominis; ****P < 0.001.

The study showed no significantly higher NRD in patients with high loudness compared to those with low loudness in SCM (*F* (2, 120) = 1.300, *P* = 0.276) (Figure 2A). Furthermore, COPD patients with high loudness exhibited significantly higher NRD in the PARA (*F* (2, 120) = 55.27, *P* < 0.001) (Figure 2B), 7thIC (*F* (2, 120) = 59.08, *P* < 0.001) (Figure 2C), and RA (*F* (2, 120) = 39.56, *P* < 0.001) (Figure 2D) when compared to those with low loudness.

### 3.3 NRD of muscles in vowel control

No differences in NRD were found for SCM (*F* (2, 120) = 0.309, *P* = 0.735), PARA (*F* (2, 120) = 0.058, *P* = 0.944), 7thIC (*F* (2, 120) = 0.051, *P* = 0.944), and RA (*F* (2, 120) = 2.128, *P* = 0.124) across vowel tasks (Table 2).

### 3.4 Borg breathlessness score in different tasks

**FIGURE 3:**
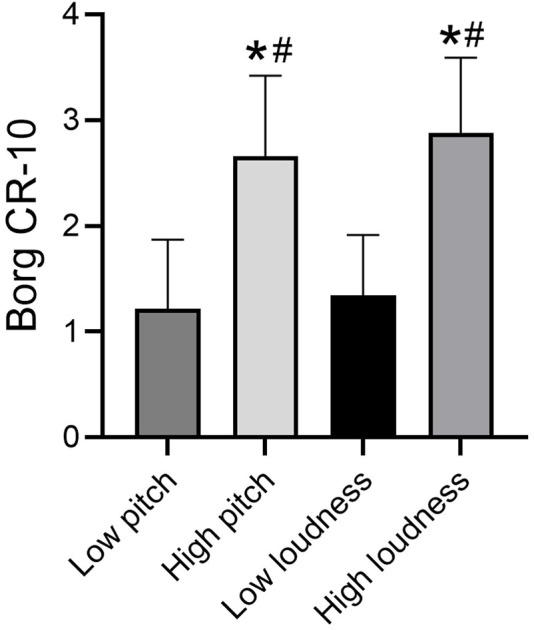
Borg breathlessness score in different tasks. Note: *compare with low loudness *P* < 0.05, ^#^compare with High loudness *P* < 0.05.

Borg dyspnea scores significantly differed across tasks [*F* (3, 160) = 66.47, *P*< 0.001]. We found no statistically significant difference in Borg dyspnea scores under low pitch and low loudness. The difference between Borg dyspnea scores under the high sound task was not statistically significant. However, Borg dyspnea scores were significantly higher after the high-loudness task than after the low-loudness task (*P*< 0.001) and after the low-pitched task (*P*< 0.001). Borg dyspnea scores were also significantly higher after the high-pitched task than after the low-loudness task (*P*< 0.001) and after the low-pitched task (*P*< 0.001) (Figure 3).

### 3.5 Correlations

**FIGURE 4:**
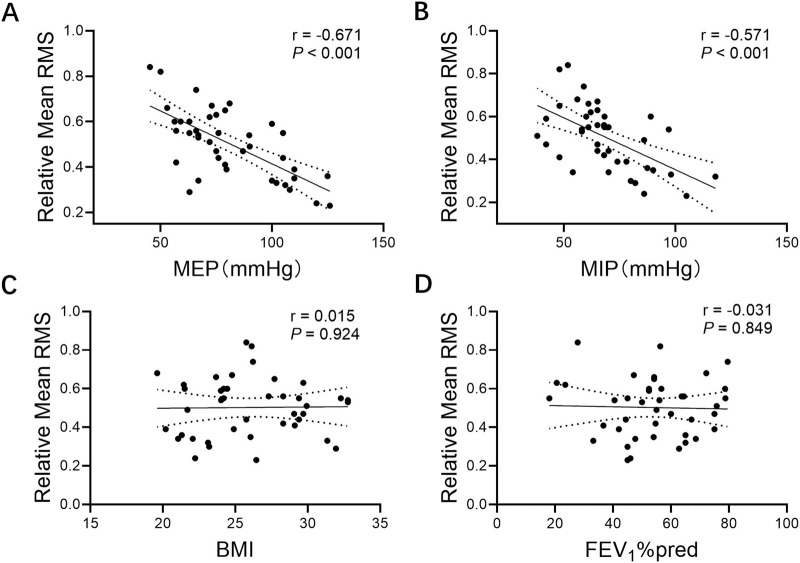
Correlations between relative mean RMS and **(A)** MEP, **(B)** MIP, **(C)** BMI, and **(D)** FEV1%pred. Note: Relative mean RMS, mean respiratory muscle RMS% MVC of parasternal intercostal muscle, seventh intercostal muscle, rectus abdominis; MEP, maximal expiratory pressure; MIP, maximal inspiratory pressure; BMI, body mass index; FEV_1_%pred, forced expiratory volume in one second % predicted.

Correlation analysis of expiratory muscle RMS measured after harmonization of pitch with baseline patient data showed that loudness was unrelated to baseline patient status. However, the RMS of the loudness control task was significantly negatively correlated with MEP (r = −0.671, *P* < 0.001) and MIP (r = −0.571, *P* < 0.001). Higher respiratory muscle strength was associated with lower RMS (Figure 4).

## 4 Discussion

Vocalization, such as singing training, can enhance expiratory muscle strength and improve lung function in patients with COPD (McNamara et al., 2017; Bonilha et al., 2009). However, its clinical effectiveness remains inconsistent and warrants further exploration due to the limited research on prescribing vocalization as a treatment (Fang et al., 2022). The neural drive of the expiratory muscles was significantly higher during high-loudness sounds compared with that during low-loudness sounds, and no differences were observed across varying pitches and vowel states. Second, high pitch and loudness produced higher dyspnea compared with low loudness and pitch. In addition, patients with higher MEP/MIP had lower respiratory muscle activation during the fixed loudness task.

### 4.1 NRD of respiratory muscle under different pitches and vowels

Categorization of vowels into high vowels and low vowels, along with mid vowels, is a well-established concept in linguistics. Subglottic pressure is produced by respiratory and laryngeal muscles, which is necessary for voice change (Traser et al., 2020). However, no significant difference was found in the NRD of respiratory muscle under different pitches and vowels. Plexico and Sandage (2012) showed that under any evaluation frequency, the threshold pressure value was not significantly different among the three consonant-vowel sequences, similar to the present results. However, Pettersen et al. found significant differences in the activation of intercostal, lateral abdominal, and rectus abdominus with different pitch and loudness levels. The vocalization task in Pettersen’s study did not control for confounding factors, such as sound loudness. By contrast, loudness was controlled in this study, which may be the reason for the difference in the results (Pettersen, 2005). In addition, high-pitched voices produced prokinetic symptoms despite no significant respiratory muscle activation. Correspondingly, Wang and Yiu (2023). Showed that the activation level of suprhyal muscle was different with different vowels due to the different shapes of the mouth and the position of the tongue. Recruitment of laryngeal muscles increased significantly with increased pitch (Zhu et al., 2022). This finding suggests that perilaryngeal muscles rather than respiratory muscles produced pitch changes. This phenomenon is consistent with the view that the original power of the respiratory muscle produces sound and that the larynx and mouth are mainly used to modify the sound (Körner and Strack, 2023; Herbst, 2017). In conclusion, this study confirms that different pitches and vowels in different training programs do not directly affect respiratory muscle training effects.

### 4.2 NRD of respiratory muscle under loudness control

The vocal effort produced significantly greater subglottic pressure during maximum-effort speech (Rosenthal et al., 2014), which may have increased the load during the expiratory phase. However, not all expiratory muscles were significantly associated with loudness. In the loudness control task, the lower ribcage embodied vocalization preferentially because only the 7thIC showed differences while PARA and RA did not. The lack of coordination of abdominal vocalization during the vocalization state in patients with chronic obstructive pulmonary disease was unexpected. We observed a more generalized chest breathing habit in patients with COPD, which may explain the lack of pronounced abdominal muscle vocalization. Some research suggests that SCM muscles may help stabilize human vocalizations (Van Houtte et al., 2013). Pettersen et al. (2005) showed that when healthy ordinary people (professional or non-professional singers) participated in vocalization training and performed vocalization content with different loudness and pitch levels, the activities of the sternocleidomastoid muscle and trapezius muscle increased; this effect was more noticeable when the respiratory demand was strong. Similarly, our observational research showed that the NRD of respiratory muscle was not significantly higher than that of low-loudness vocalizations in SCM in patients with COPD.

### 4.3 Clinical implications

Previous studies have shown that comprehensive pulmonary rehabilitation, based on standard protocols, can improve dyspnea and quality of life in patients (Troosters et al., 2023). However, there are still inconsistencies in vocalization training programs for COPD patients (Fang et al., 2022). This study offers clinical implications for the implementation of vocalization training. Regarding the physiological effects on patients, Fu et al. showed that collective singing and vocal training improved the maximum expiratory pressure and exercise ability of older people in the community (Fu et al., 2018). Lord et al. (2012) reported that the objective physiological state of patients with COPD did not change after vocalization training. However, the content and intensity of singing are not disclosed in vocalization studies, which may be the reason for the differences in the outcome indicators (Patel et al., 2022; Selickman and Marini, 2022). In addition, correlation analysis showed that vocalization of the same loudness produced great stimulation in patients with low respiratory muscle strength. This finding is consistent with the theory that the intercostal and abdominal muscles provide vocal expiratory support (Pettersen et al., 2005; De Troyer and Boriek, 2011). Muscle control and improvement are required to ensure an intensity threshold for training; according to the results of this study, vocalization at a lower intensity may not be sufficient to engage the respiratory muscles fully. At the same time, the degree of dyspnea after the high-loudness task was higher than that after the high-pitched task, while the degree of dyspnea between the low-loudness task and the low-pitched task was not significantly different. Hence, home oxygen therapy, breathing exercises, and other treatments that can improve dyspnea can be combined with vocalization training for improved clinical outcomes. Personalized vocalization prescriptions for patients with COPD should consider the respiratory muscle state of patients rather than simply pursuing vocalization loudness.

### 4.4 Limitation

Although the study examined the NRD of respiratory muscles to different vocalization tasks, this study still has some limitations. First, this study only addressed the physiological effects of vocal tasks and did not address the psychological effects of vocal training as an artistic engagement. Second, all syllable pitches and loudness are difficult to study because of the patients’ limited tolerance. Relatively simple /a/, /i/, and/u/were chosen for this study, considering vocal teachers’ suggestions and previous studies. Finally, participants included patients with COPD only who had performed primary vocal exercises to study the effect of training on uninitiated patients. Therefore, the results of the article cannot be generalized to patients who have received full vocalization training.

## 5 Conclusion

In vocalization training for patients with COPD, focusing on increasing loudness, rather than pitch or vowel, led to the activation of the expiratory muscles. High pitches and loudness produced dyspnea compared with low pitches and low loudness. The maximal expiratory/inspiratory pressure was negatively correlated with respiratory muscle NRD in the same loudness vocalization task.

## Data Availability

The original contributions presented in the study are included in the article/supplementary material, further inquiries can be directed to the corresponding author.
